# Parental training in groups: a brief health promotion program

**DOI:** 10.47626/2237-6089-2019-0055

**Published:** 2021-02-26

**Authors:** Marcella Cassiano Russo, Isabela Pizzarro Rebessi, Carmem Beatriz Neufeld

**Affiliations:** 1 Universidade de São PauloRibeirão PretoSPBrazil Universidade de São Paulo (USP), Ribeirão Preto , SP , Brazil .

**Keywords:** Cognitive-behavioral therapy, educational practices, health promotion, groups, parental training

## Abstract

**Objective:**

To propose a brief parenting program offered in the context of health promotion and evaluate the immediate results relating to use of appropriate parenting practices and quality of parent-child interaction.

**Methods:**

Forty-five parents of school-age children from two non-governmental institutions located in a medium-sized city in the state of Sao Paulo participated in the study. The following assessment tools were used in the pre and post-tests: the Child Behavior Checklist (CBCL), Quality of Family Interaction Scales (EQIFs), and the Brazilian Economic Classification Criteria (CCEB). Only scores of parents who attended 75% of the program were included in the analysis (25 participants).

**Results:**

Most of the participants who completed the program were grouped in socioeconomic levels B and C (72%) and the complaints reported in the pre-test centered on disobedience and stubbornness (29.6%, each). Regarding parents’ perceptions of their educational practices and interaction with the children, improvements were detected in several of the aspects measured: affective relationship, involvement, model, communication, rules and monitoring, and children’s feelings, besides reduction in use of physical punishment and negative marital atmosphere (p < 0.03). Reductions were detected in aggressive behavior (p = 0.02) and externalizing problems (p = 0.04).

**Conclusion:**

Despite the small sample and application in a specific community, this quick and affordable intervention seems to have yielded improvements in parent’s monitoring and their affective relationships with their children, in addition to reductions in punishments and children’s aggressive behavior, contributing to better parent-child interaction in the community.

## Introduction

The family is the fundamental unit of society responsible for educating and socializing children. It is through interaction with their parents that children begin to establish emotional bonds, create their first cognitive and relational schemes, come into contact with behavioral and language rules and are also gradually introduced to a larger social environment. ^1 - 3^

Several factors can hinder child development and contribute to the emergence of psychopathologies, including deficits in parental skill repertoire, inconsistent discipline, lack of monitoring, and excessive punishment. Some other parent-specific problems can also have effects, such as marital conflicts, difficult parental mood, parental emotional adjustment problems, and maternal depression and anxiety. ^4 , 5^

Despite the importance of parents in child development, they rarely receive guidance on how to educate their children. Their parenting style is usually based on their experience and trial and error. ^6 , 7^ It has been observed that the prevalence of infant social-emotional and behavioral problems is increasing, especially at school age. ^8^ This situation requires rapid and effective strategies to identify problems and initiate effective treatments, which are more economically viable than late interventions. ^1 - 9^

A total of 13-20% of children living in the United States experience problems with cognitive, social, and emotional development and receive treatment for clinically significant disorders late. A global epidemiological survey showed that symptoms only improved without any specific treatment in half of children who exhibited significant problems in pre-school and early school ages. As time passed and responsibilities increase, others tended to face more serious problems, such as truancy and difficulties with family and with peers, in addition to increased risk of substance abuse in adolescence and adulthood, with a considerable impact on their families and on society, as well as higher treatment costs, which are not affordable for all families. ^9^

In Brazil, an epidemiological survey ^10^ estimated rates of child and adolescent mental disorders at around 10-20%. Among problems diagnosed, the highest prevalence rate was 4.4-7% for externalizing defiant disorder and conduct disorder. It has been hypothesized that disorders at subclinical levels could significantly increase these percentages. ^11 , 12^

Due to the early influence of parents on child development and socialization, some interventions have been designed to help parents develop optimum strategies for coping with their children’s emotional behavior. Parental training is the most common behavioral intervention with efficacy for treatment and prevention of various psychological problems in children. ^13 - 15^ The results suggest improvement in children’s behavior, acquisition of social skills, and increased treatment effectiveness in cases with concomitant interventions.

There are several evidence-based parental training programs available in the literature. ^16 , 17^ Most of these programs target families that need to manage problems with children’s behavior. In recent decades, some support has been given to preventive family interventions as well as programs aimed at promoting family health, focusing on better interactions between parents and children. ^1^

These health promotion programs are also intended to prevent emotional and behavioral problems in children. Their application has yielded positive results in educational practices and family interaction. ^18 - 20^ Unfortunately, they are long-term programs (at least 8 sessions lasting 2 hours each), making it difficult to introduce them in the Brazilian public health context and making them inaccessible to low-income populations. Intervention duration is a positive factor for participant adherence, which is always a challenge in parental training, especially in preventive interventions and health promotion. ^21^ Current literature increasingly describes investment in brief evidence-based programs to promote positive relationships between parents and children, ^1 - 22^ especially for populations in vulnerable places, with limited access to health care and preventive programs. ^15 , 23^

In an attempt to include families with lower socioeconomic levels ^24^ some interventions have been created that are designed to be applied in Brazilian schools and social institutions, as well as assessment tools to measure improvement in the participants and their children. Likewise, most of these training programs are based on behavioral approaches and provide guidance for parents on how to observe and modify child behavioral problems. However, there are other fundamental aspects that do not receive as much attention, such as emotion and cognition. The way parents perceive their own parental skills is important for regulation of their feelings and behaviors. When parents realize they are competent in their roles as parents, they become more consistent, confident, and resilient when facing challenges and, therefore, their relationships with their children improve, contributing to their mental health. ^25^ With regards to inclusion of the cognitive aspect in parental training, it was observed that identification of parents’ beliefs about their roles as educators and the impact of educational practices on children’s behavior can benefit learning and establishment of appropriate practices. ^14^

The PROPAIS-USP program (Programa Cognitivo-Comportamental de Orientação de Pais visando à Promoção de Saúde, or simply PROPAIS I ^26^ ) also contributed to inclusion of cognitive aspects in parental training, focusing on possible ideas that can derive from educational practices and on restructuring those that are distorted, with direct impact on parents’ educational practices. Subsequently, the PROPAIS II program was proposed, based on PROPAIS I. This was a brief, heterogeneous parental training program focused on health promotion. It is a 6-session program, with 1-hour group meetings held in schools and community centers in neighborhoods with a high degree of social vulnerability. It provides basic guidelines for development of more adequate educational practices and protective family interaction. ^27^

The aim of this study is to report on the implementation and results of the preliminary program for parents of children from two low-income communities in Ribeirao Preto, SP, Brazil. This article analyzes the socioeconomic characteristics of the sample and results related to the parents’ perception of children’s behavioral problems and their own use of parenting skills following the intervention.

## Method

### Participants

The study sample comprised 25 parents of school-age children who had at least one child aged between 6 and 14 years old enrolled in school. Participants were considered to have completed the program if they attended at least 75% of the sessions. The mean age of parents was 37.37 years (standard deviation [SD] = 8.5) and the mean age of their children was 9.05 years (SD = 1.9), 56% (n = 14) of whom were girls. Participation of mothers was more prevalent, accounting for 75% of the sample (n = 20), followed by grandparents (12%, n = 3), and fathers (8% n = 2). Most of the parents were married or living together (80%, n = 20); only one participant was single, and four were divorced/separated. The number of children under the responsibility of each participant was as follows: 48% of the sample (n = 12) were responsible for two children, 24% (n = 6) for one child, 24% (n = 6) for three children, and 4% (n = 1) for four children.

Another 20 parents declined the intervention after the pre-test or after the first session. Among these, 85% (n = 17) were mothers and 5% were fathers, grandparents, and aunts (n = 3 for each). Most of them (85%, n = 17) were married/living together. Two participants were divorced (10%) and one was single (5%). The mean age of the participants in this group was 35.6 (SD = 8.2). The mean age of the children was 8.85 years (SD = 2.54) and 55% (n = 11) of them were girls. Although the characterization data of the participants who dropped out of the intervention might be useful to indicate reasons for their withdrawal, these parents were not included in the study data because they did not have the minimum adherence required.

All of the 45 participants were informed about the study goals and were asked to sign an informed consent form. Parents were invited to take part at a parents meeting that the institutions hold for parents at the beginning of each school year. The protocol was introduced to the institution and, after they expressed interest, the therapist asked for space at parent meetings and went to introduce the protocol with the interns. Parents who expressed interest in participating and had children at the right age (and remained assiduous at the pretest sessions) made up the group. All procedures were conducted in compliance with Brazilian National Health Council Resolution no. 196/1996 and the Helsinki Declaration. The study protocol was approved by the research ethics committee of the university (USP). The samples were selected by convenience.

### Instruments

In the pre-test, the main difficulties parents faced concerning their child’s upbringing were recorded using a short form. Families’ socioeconomic status (SES) was measured using the Brazilian Economic Classification Criterion (CCEB). ^28^ This criterion classifies socioeconomic status by assigning scores to the number of household assets and the head of the family’s educational level. It comprises five levels: A is the highest and E the lowest.

Parent’s perceived educational practices and quality of family interaction were evaluated using the Quality of Family Interaction Scale (EQIF-parent version). ^29^ This contains 10 items rated on a 5-point Likert scale with a maximum score of 52. Seven items were used to assess the positive aspects of parent-child relations: affective relationship, involvement, rules and monitoring, positive communication with children, parental model, positive marital atmosphere, and children’s feelings. The three remaining items assess the frequency of negative practices: physical punishment, negative communication, and negative marital atmosphere.

Parents’ perceptions of the children’s behavioral and emotional problems were assessed using the Child Behavior Checklist (CBCL). ^30^ This checklist evaluates children’s social competence with 20 items scored on a 4-point Likert scale and also measures internalizing and externalizing behavioral problems using a 3-point Likert scale.

### Procedures

The initial forty-five participants were allocated to six parental training groups, with an average of 5 participants per group. The PROPAIS II intervention took place at two nongovernmental educational institutions located in socially vulnerable neighborhoods. Four groups were formed in a center for strengthening bonds (Institution I) and two groups in a center for supplementary educational activities (Institution II). The program was delivered by two therapists who had been trained in the PROPAIS II protocol. Sessions lasted 1 hour each and participation was voluntary after parents expressed interest at the institutions’ regular meetings. These six groups enrolled the first participants to express their interest to the director of each institution. Participants who completed the intervention were evaluated before and afterwards and parents who dropped out from the intervention were only evaluated with the pre-test. A linear mixed-effect model (random and fixed effects) was used to test for possible differences between the groups. Since this did not indicate differences, the participants were analyzed together. The pre and post-test EQIF scores were compared with the Wilcoxon test using the Statistical Package for the Social Sciences (SPSS).

The intervention schedule is shown in Table 1 .

**Table 1:** General structure of the PROPAIS II intervention sessions

Sessions	Main activities
Pre-test	Administration of the EQIF, CBCL, and CCEB instruments
	Establishing bonds and group cohesion
1st and 2nd	Survey of difficulties, profile identification, and ways to educate
	Psychoeducation of parental education social skills and child development
	Psychoeducation and observation practice of behavior rules and effective communication techniques
3rd and 4th	Self-monitoring practice (behaviors and emotions)
	Initial psychoeducation according to Beck’s cognitive model
	Cognitive model and identification of cognitive distortions in education
5th and 6th	Management of uncomfortable emotions and relaxation techniques
	Problem-solving in family daily life
Post-test	Re-administration of the instruments

CBCL = Child Behavior Checklist; CCEB = Brazilian Economic Classification Criterion; EQIF = Quality of Family Interaction Scale.

Adapted from Cassiano and Neufeld. ^27^

The program consists of six sessions focusing on positive parental educational practices and social skills targeting better parent-child interaction. ^27^ Although the sessions were structured, there was flexibility for some changes to fit the needs and characteristics of the participants.

## Results

### Characterization of participants’ families and initial complaints

In terms of their family structure characteristics, 84% of participants (n = 21) were members of nuclear families, 8% (n = 2) were members of blended families (stepfather/stepmother and half-brothers), and 8% (n = 2) were families headed by grandparents living with their sons/daughters and their grandchildren in the same residence.

The parents were classified into the following SES categories: C (36%, n = 9), B2 (28%, n = 7), D (28%, n = 7); B1 (8%, n = 2); one participant in group 4 and one in group 6 were classified as category D, held at Institution II. The main complaints raised by the participants in the pre-test were disobedience and stubbornness (29.6%, n = 8 each). During the sessions, parents complained about their children being “rude,” “having no limits,” and being “agitated” and “lazy,” but these complaints were not mentioned during the pre-test.

### Parental educational practices and quality of family interaction

These results are shown in Table 2 .

**Table 2:** Comparison between mean pre-test and post-test scores for educational practices and family interaction

Variable	Pre-test Mean (SD)	Post-test Mean (SD)	Comparison p	Wilcoxon Z score
Affective relationship	30.12 (4.50)	37.56 (2.41)	0.00*	-4.377 ^†^
Involvement	36.12 (3.57)	37.76 (2.18)	0.01*	-2.653 ^†^
Rules and monitoring	27.52 (2.12)	28.80 (1.70)	0.01*	-2.633 ^†^
Physical punishment	10.16 (2.83)	7.84 (0.99)	0.00*	-3.248 ^‡^
Positive communication	10.60 (2.18)	12.64 (2.32)	0.00*	-3.323 ^†^
Negative communication	11.28 (2.50)	9.48 (2.65)	0.01*	-2.586 ^‡^
Positive marital atmosphere	15.44 (5.77)	16.44 (8.30)	0.24	-1.171 ^†^
Negative marital atmosphere	11.16 (3.64)	7.08 (3.69)	0.00*	-3.858 ^‡^
Model	12.00 (2.02)	13.16 (1.67)	0.03*	-2.247 ^†^
Children’s feelings	26.16 (2.69)	27.16 (2.19)	0.02*	-2.274 ^†^
Total score	1.25 E2 (13.73)	1.49 E2 (14.41)	0.00*	-4.252 ^†^

SD = standard deviation.

* p < 0.05.

^†^ Based on negative ranks.

^‡^ Based on positive ranks.

Statistical differences were found for total EQIF score (W = -4.25, p = 0.00) and most items (W < -2.25, p < 0.02). No difference was found for Positive Marital Atmosphere (W = 1.71, p = 0.24), which evaluates good interaction between spouses (such as compliments, expressions of affection and appropriate communication).

### Parents’ perceptions of behavioral problems

The six groups were treated together since the Kruskal-Wallis did not detect any differences. The Wilcoxon test (W) was used to compare CBCL scores before and after the intervention.

The results are shown in Table 3 .

**Table 3:** Comparison between mean pre-test and post-test scores for child behavior from the Child Behavior Checklist

Variable	Pre-test Mean (SD)	Post-test Mean (SD)	Comparison p	Wilcoxon Z score
Activity	36.07 (7.35)	37.60 (6.84)	0.22	-1.220 ^†^
Social	42.64 (9.26)	42.96 (11.02)	0.76	-0.310 ^‡^
School	41.80 (8.39)	43.25 (9.14)	0.53	-0.625 ^†^
Total competence	36.57 (12.95)	36.55 (8.64)	0.66	-0.443 ^†^
Anxiety and depression	61.84 (10.02)	58.36 (8.03)	0.10	-1.637 ^‡^
Withdrawal	60.68 (8.24)	59.68 (7.47)	0.49	-0.684 ^‡^
Somatic complaints	59.92 (8.82)	57.24 (7.70)	0.13	-1.535 ^‡^
Social problems	59.80 (7.65)	58.40 (5.64)	0.45	-0.746 ^‡^
Thought problems	58.80 (9.35)	56.60 (9.03)	0.20	-1.288 ^‡^
Attention problems	60.80 (11.22)	60.08 (12.12)	0.48	-0.715 ^‡^
Rule-breaking problems	58.00 (6.95)	57.96 (6.94)	0.73	-0.341 ^‡^
Aggressive behavior	64.04 (10.22)	59.28 (10.16)	0.02*	-2.324 ^‡^
Internalizing problems	61.60 (11.07)	58.44 (8.64)	0.15	-1.443 ^‡^
Externalizing problems	61.96 (8.74)	58.84 (9.91)	0.04*	-2.040 ^‡^
Total problems	62.68 (9.46)	58.84 (9.91)	0.11	-1.586 ^‡^
Affective problems	63.32 (7.15)	61.20 (8.44)	0.13	-1.497 ^‡^
Anxiety problems	60.52 (9.08)	58.40 (6.79)	0.25	-1.050 ^‡^
Somatic problems	58.32 (9.71)	59.60 (9.90)	0.21	-1.247 ^‡^
Attention and hyperactivity	62.24 (9.03)	57.76 (7.38)	0.21	-1.251 ^‡^
Oppositional defiant	60.40 (7.18)	57.84 (6.24)	0.06	-1.880 ^‡^
Behavior problems	60.00 (8.46)	54.20 (6.48)	0.21	-1.252 ^‡^
Slow cognitive time	55.60 (6.97)	55.60 (13.97)	0.49	-0.699 ^‡^
Obsessive problems	59.96 (10.56)	58.48 (8.30)	0.18	-1.328 ^‡^
Post-traumatic stress	61.80 (9.49)	36.55 (8.64)	0.14	-1.478 ^‡^

SD = standard deviation.

* p < 0.05.

^†^ Based on negative scores.

^‡^ Based on positive scores.

Statistical differences were found in the variables aggressive behavior (W = -2.32, p = 0.02) and externalizing problems (W = -2.04 p = 0.04) with lower scores after the intervention. No changes were detected in the variables related to other externalizing and internalizing behaviors or in children’s social competence (W > -0.310, p > 0.06).

In addition to the analyses comparing participants’ scores before and after the intervention, participants’ pre-test and post-test results were also used to categorize them according to the clinical severity of their children’s problems. The CBCL categorizes children as clinical, borderline, or non-clinical, but the first two categories were grouped together in this study and data are only presented for non-clinical and clinical categories.

Pre-test and post-test severities were analyzed for the following variables: social competence, internalizing problems, and externalizing problems. The frequencies of each severity classification are shown in Table 4 .

**Table 4:** Frequency of clinical and non-clinical severities for the variables Total Competence, Internalizing Problems, and Externalizing Problems in pre-test and post-test

	Social competence		Internalizing problems		Externalizing problems	
Tests	Pre	Post	Pre	Post	Pre	Post
Clinical	15	13	16	11	16	11
Non-clinical	4	6	9	14	9	14
Missing	6	6	0	0	0	0

## Discussion

In Brazil, there is considerable need for early family intervention with the purpose of identifying and treating early childhood problems, especially among needy families with little access to health care and education on how to manage children’s behavioral problems. Despite this, there are few programs that can address this specific population, making intervention services accessible to communities in schools and basic health units. ^1 - 31^

In response to this demand, we proposed the PROPAIS II for areas of high social vulnerability and low income, delivered through institutions that offer protection to children and families. The CCEB identified the distribution of participants’ social status as ranging from D (low income) to B1 (upper middle class), with a prevalence of middle class participants (C and B2). Although the CCEB identified characteristics of the middle class, these parents and children were living in a socially vulnerable area, where access to additional educational opportunities for their children is problematic.

Participation of mothers in the groups predominated, as has been reported in another international program ^17^ and in national parental training programs. ^23 , 26 , 32^ No significant differences related to the ages of children and parents and their complaints were found in this study, when compared with other parental training programs. Analysis of the characteristics of parents who completed the intervention and parents who quit showed that the age and sex distributions of parents and children were very similar in both groups. Therefore, there was no evidence of differences in characteristics that could explain the dropout rates.

The problem of parents’ adherence to parental training programs, particularly for health promotion, is not specific to PROPAIS II and has been widely described in national and international literature, along with suggestions for measures to minimize it. These include publicizing programs in several different media, easy access to the location for parents, enabling monitoring of their children during sessions, individual sessions with parents to raise issues causing greatest difficulties with following the sessions, telephone contact or electronic messages from therapists before sessions, and provision of snacks during the course of the interventions. ^3 , 16 , 32 - 35^

With the exception of publicity in several media, which was not applicable since the PROPAIS II intervention was only offered at two institutions in this study, all of the measures were incorporated into the sessions. Despite this, adherence was not totally satisfactory. In literature, some authors point out that some other features can be used in an attempt to improve rates, such as dramatizations of everyday life, audio and video materials, and multidisciplinary training involving parents, children, and schools. ^36 - 38^ The last two points could not be incorporated into the PROPAIS II for budgetary reasons. It is a real challenge to manage to offer an affordable parental training program with all the resources necessary to maintain attendance, especially in Brazil.

### Quality in family interaction and educational practices

The post-intervention results were highly significant for quality of family interaction and parental educational practices; we observed improvements in educational practices. Aspects of marital relationship such as communication and expression of affection remained unchanged.

The results were consistent with those reported by international parental training programs aimed at health promotion. The most important changes in the literature indicate positive improvement in monitoring, in consistency in applying rules, and in reducing physical punishment, and a better parent-child relationship. ^18 , 22 , 39^

The mean pre-test EQIF scores were high for positive subscales and increased even further in the post-test. For the negative subscales, the means started low, and similarly, decreased further in the post-test, which may indicate that parents did not seem to face many difficulties in maintaining positive interaction with their children and in avoiding coercive education before the intervention.

It is important to note that the participants did not seek the intervention because of clinical complaints, since this was a community sample, and so the initial intervention scores were expected to be reasonable or good. Another Brazilian health promotion study ^24^ reported adequate scores for parental education practices before intervention, but there was nevertheless a significant improvement in parents’ educational practices and interaction with their children. It seems that those individuals who agreed to participate in a preventive intervention and health promotion program already had a greater repertoire of appropriate educational practices. Notwithstanding, even if parents already have a good repertoire of educational practices, it seems that health promotion programs can still help improve these practices, which is consistent with the goals of health promotion.

Lower negative scores and higher positive scores observed after application of PROPAIS II could suggest changes in the way these participants evaluate their educational practices and how they interact with their children. This aspect was more evident in affective relationship scores. Affection was a theme highlighted in the program and was remembered by all participants in the final evaluation as a key element for implementation of changes in the forms of educating children. Encouraging use of positive educational practices, such as praise, has been a prominent theme in parental training programs. Emphasis of positive qualities is a good strategy for promoting self-esteem and empathy in children, contributing to their relationships with others. ^40^

In all sessions, our program emphasizes parents’ observations of their own behavior, emotions, and thoughts during interactions with their children. All examples of situations used for training of praise and reinforcement of good behavior were provided by the parents and guided by the therapists. This may have contributed to better discrimination of moments worthy of praise and positive attention and higher frequency of adoption of these habits by the participants. It may also have contributed to the fact that parents were more aware of changes in their own behavior than changes in their children’s behavior. Since the program focuses on parents’ behavior, it may be more difficult for them to perceive changes in their children’s behavior and easier to perceive changes in their own. It is more important to tell parents to praise their children at the right moments than to teach them how to praise their children. When praise is not used at the opportune moment, it becomes less effective and may have a negative impact on motivation and children’s behavior. During the course of the program, the parents were instructed to detect and select important practices and times for praise, as well as other reinforcements that should be used in addition to compliments, such as spending quality time with the child, for example.

The higher scores in the subscales involvement, feelings of parents towards their children, and rules and monitoring may be an indication of closeness between parents and children, such as greater patience to explain and to define rules and awareness that change is a difficult process that requires consistency and persistence. The increase in the model subscale score might indicate that participants became more aware that their own behaviors were being observed and replicated by children. The importance of parents as models was highlighted in program and some suggestions for daily behavioral changes were elicited and taught by therapists.

A high score in positive communication and a low score in negative communication (screaming, scolding, and labeling) were observed in the post-test. This improvement may indicate a change in children’s behavior, but could also indicate an increase in parental awareness about the importance of better communication and assertiveness in the education of their children.

Reduced physical punishment and negative marital atmosphere scores were also significant. Despite using different measurement instruments, other parental training programs have also observed reduced physical punishment, a topic that has been the focus in all programs available in the national and international literature. ^23 , 33^ One of the programs that used the same measurement instrument obtained similar behavioral changes, including greater participation and involvement, clarity and consistency in setting rules, use of praise and less use of physical punishment. ^23^ With similar goals, both programs obtained great results regarding the behavioral measures; the main differences between them were the shorter duration of the PROPAIS II and the cognitive-behavioral approach.

### Children’s behavior

The pre-test CBCL scores showed various behavioral problems and more children were classified based on their clinical conditions than on normality. Since this was a community sample, this characterization of the children’s behavior was a major concern, considering that the scores were similar to those for clinical samples of children undergoing psychological care. ^41^ These indices corroborate the need for interventions that can identify problems early and help families to cope with them.

After application of PROPAIS II, we observed lower scores for behavioral problems and changes from the clinical to non-clinical category in three variables: social, internalizing, and externalizing problems. There was also improvement in some behavioral problems allocated to the category externalizing problems as a whole, particularly aggressive behavior. The children’s behavior was not observed directly. Studies based on parents’ perceptions may indicate that children showed improvement in a certain behavior previously considered a problem, but also that parents have begun to interpret their children’s behavior differently. ^33^ These hypotheses are not mutually exclusive. We emphasize that, regardless which of explanation is more likely, in programs that use this type of evaluation, the effect of changes on the daily lives of families tends to be positive: either in terms of lower incidence of child problems or in terms of flexibility of parental perception about the problems and their causes. Since PROPAIS II is a health promotion program, these results are consistent with its goals, which are to promote health and well-being in those communities that, in many cases, do not have access to many interventions.

No changes were detected in children’s internalizing behaviors after the PROPAIS II or were described in studies of other preventive parental training programs. We hypothesize that the internalizing behavioral problems were not only less prevalent in the population, but were also difficult for parents and teachers to detect. Studies have shown that parental training is the best modality for treating disruptive disorders in children. As much as PROPAIS II aims to deal with different complaints, the emphasis given by participants on externalizing problems expressed in CBCL, on the registration form, and in the session reports led to greater efforts to minimize them. This may explain why this and other programs that focus on changing children’s behavior obtain more significant results in reducing externalizing problems than internalizing ones. This could also explain why the changes in internalizing behaviors were not detected despite parents perceiving improvement in their educational practices and communication with their children.

Another aspect that may have influenced the maintenance of several behavioral problems in children that were detected in the pre-test was the short duration of the program. A change in behavior tends to take considerable time and be may not be achievable during a very brief intervention.

The cognitive changes in the participants, their perceptions of the adequacy of educational practices, and their impact on children’s behavior could not be quantitatively measured in this study, due to the absence of instruments to assess the cognitive aspects involved in educational practices. Recently, a cognitive scale named “Me as a Parent” was developed by Australian researchers ^25^ to measure parenting sense of competence with the following characteristics: self-efficiency, self-sufficiency, and self-management. If validated and translated for use in Brazil, it could surely contribute to quantitative assessment of the cognitive results of PROPAIS II in future applications of this training program.

## Final considerations

The results of the initial implementation of PROPAIS II showed significant improvements, mainly related to parental educational practices and parent-child interaction. The greatest difficulty in the study was initial adherence to the program. We tried to directly understand the reasons for loss of interest by the participants who were invited to come to the meeting, but attempts to discover reasons via telephone failed.

The difficulty with attendance may be related to the fact that participants were not necessarily looking for treatment. In Brazil, it is not very common that parents are involved in volunteering activities in schools and other children’s institutions. Low parental involvement may be due to excessive work, lack of time, and a shortage of events that include families in institutions, making it difficult for them to appropriate a space that they only recognize as a place for children. Such assumptions may explain the difficulties encountered in this study and in similar programs.

It is important to continue to invest in strategies to maintain participants’ attendance, such as publicity on media, direct contact with the institutions, opportunities to resolve doubts raised by parents, simple content, and a focus on practice, among others. While planning each intervention, it is also important to recognize the demand of participants within an institution so that the proposed program can address the real needs of families. Therefore, therapists, parents and institutions should work together and the characteristics of the community should be considered.

Despite limitations, the PROPAIS II yielded positive changes in parental behaviors, contributing to clinical practice and to research in parental training. It is a successful health promotion program design for parents that is quick and easy to use, making it accessible for replication and enabling its use with families of children without apparent clinical demands, which could act as a protective factor for child development and better family interaction. In particular because it accessed communities with high social vulnerability and low income, PROPAIS II showed a great cost benefit. It would be of great benefit to adopt public polices designed to increase parental support in schools and in the community, with partnerships between parents and schools and parental social engagement.

For further studies, follow-up and supplementary evaluation measures in addition to the self-report instruments are essential to evaluate the extent and duration of the observed changes. Investment in instruments to measure cognitive aspects of parenting practices is very important to assess whether inclusion of cognitive components in the program really helps to maintain the behavioral changes. Additionally, expansion of the sample and implementation of programs in other settings where low-income families can attend is crucial.
